# Effect of maximal mental effort during elastic band training on neuromuscular adaptations in older women

**DOI:** 10.3389/fragi.2025.1662126

**Published:** 2025-11-19

**Authors:** Lacey Harper, Kayla Anderson, William Reed, Kaden Buford, Anna Acosta, Jacob Grazer, Robert Buresh, Tim Martin, Garrett Hester

**Affiliations:** 1 Exercise Science and Sport Management, Kennesaw State University Wellstar College of Health and Human Services, Kennesaw, GA, United States; 2 Department of Psychological Sciences, Kennesaw State University Radow College of Humanities and Social Sciences, Kennesaw, GA, United States

**Keywords:** sarcopenia, voluntary activation, hypertrophy, strength training, aging

## Abstract

**Introduction:**

Neural impairments contribute to age-related weakness, yet strategies complementing practical exercise training to target neuromuscular adaptations are lacking.

**Objective:**

This study aimed to determine if combining maximal mental effort (MME) with elastic band training (EBT) augments neuromuscular adaptions in older women.

**Methods:**

Untrained older women (71 ± 4 years) were randomly assigned to EBT (n = 11), EBT + MME (n = 13), or a control (CON; n = 10) group. EBT and EBT + MME performed 6 weeks of moderate-intensity, total body elastic band training, but EBT + MME imagined a maximal muscle contraction during each exercise. Neuromuscular outcomes including voluntary activation (VA), contractile properties, dynamic strength (1-repetition maximum; 1-RM), and isometric peak torque of the elbow flexors (EF) and knee extensors (KE) were assessed. Additionally, KE and EF cross-sectional area (CSA) and muscle quality were captured, as well as lean mass. Two-way mixed ANOVAs were used to compare groups across time.

**Results:**

VA (p = 0.182) and contractile properties (p > 0.05 for all) remained unchanged. Compared to CON, 1-RM (p = 0.004), KE peak torque (p = 0.034), CSA (p < 0.001 for both), and muscle quality (p = 0.001–0.021) improved in EBT and EBT + MME, but no differences existed between these groups (p > 0.05). Lean mass remained unchanged (p = 0.481–0.753).

**Conclusion:**

Compared to EBT, MME did not result in augmented strength or VA. Future research is needed in sarcopenic or older adults suffering from greater age-related weakness. Despite the short training duration, positive effects of home-based, EBT were found for muscle size, quality, and strength in older women. The promotion of accessible forms of resistance training for older adults, such as EBT, is warranted.

## 1 Introduction

A leading health issue estimated to effect up to 40% of older adults is sarcopenia, the age-related loss of muscle mass and function (Mayhew et al., 2018). Despite muscle atrophy traditionally being considered the culprit for age-related weakness, the critical influence of neural decrements is becoming increasingly clear. Age-related reductions in muscle strength are 2–5 times greater than that of muscle size in older adults, indicating that muscle atrophy only modestly explains the decrease in strength (Delmonico et al., 2009). The discrepancy between age-related loss in muscle mass and strength is at least partially explained by the inability of the central nervous system to voluntarily activate skeletal muscle in older adults (Clark et al., 2019; Yue et al., 1999; Morse et al., 2004). Recently, it was reported that neural excitability explained ∼33% of the variability in strength in older adults with clinically meaningful weakness, roughly equal to that explained by lean mass (Clark et al., 2021). The significant influence of nervous system decrements on age-related reduction in muscle function underscores the need for novel strategies that target neuromuscular adaptations.

A limited body of work indicates that using a maximal mental effort (MME), the cognitive demand associated with an intended action, with resistance training (RT) may enhance neural mechanisms underlying strength production (Jiang et al., 2016). MME involves mentally urging the agonist muscle to maximally contract during submaximal RT without attempting to increase contraction speed. In older adults, a high-intensity isometric RT group was compared to a low-intensity (∼30% maximum strength) group that used MME by mentally urging their muscle to contract maximally during training (Jiang et al., 2016). Similar strength gains were found between both groups, despite the substantial discrepancy in training intensity. Strength gains were accompanied by increased motor activity-related cortical potentials in the MME group only, suggesting improved cortical drive to the muscle. Another study demonstrated that low-intensity RT combined with MME improved strength in young adults, whereas low-intensity RT alone did not, signifying that MME had a supplementary effect on strength (Jiang et al., 2017). MME may be a promising strategy in older adults to augment strength adaptations following RT by targeting neural mechanisms. However, to date, the few studies examining the synergistic effect of RT and MME are limited to training of a single muscle group with isometric contractions in a laboratory setting (Jiang et al., 2016; Jiang et al., 2017).

It is critical to determine whether MME combined with practical, moderate-intensity RT yields a synergistic effect on muscle strength compared to moderate-intensity RT alone. Given the low participation in RT (∼20%) (Keadle et al., 2016) and reported barriers (Burton et al., 2017; Van Roie et al., 2015) by older adults, we employed elastic band resistance training (EBT) due to its accessibility and effectiveness for increasing muscle strength and endurance, functional capacity, and body composition in older women (Colado and Triplett, 2008; Liao et al., 2018). It is warranted to determine efficacy of combining MME with EBT in older women since, despite living longer, they demonstrate greater functional limitations and incidence of frailty than men (Gordon et al., 2017). Thus, the purpose of this study was to determine if combining MME with EBT augments neuromuscular adaptions in older women. We hypothesized that utilizing MME during EBT would result in greater improvements in strength and voluntary activation compared to EBT alone. It was also hypothesized that improvementsin muscle size and quality, if any, would be similar for EBT with and without MME. This was based on the short duration of training (6 weeks) and expectation that any differences in stimuli would be limited to the nervous system. Additionally, given the cognitive demand of MME, it is important monitor affective responses to yield insight related to motivation or barriers to this intervention.

## 2 Materials and methods

### 2.1 Participants

Fifty-two community-dwelling women aged 65–79 years were recruited for this study. Of the 52 participants recruited, 34 participants completed the study (Supplementary Figure S1). G∗Power (v. 3.0.10) software indicated a total sample size of 42 would detect a medium effect size (*f* ​ = ​0.25) with a statistical power of 0.80 for a two-way analysis of variance. Individuals were included if they had not engaged in structured aerobic or resistance training for 3 years, were not reliant upon a walking aid, and reported being able to rise from a chair unassisted. Individuals were excluded if they had any chronic diseases that contraindicate exercise participation. Additional exclusion criteria included uncontrolled hypertension, musculoskeletal injury in the past 6 months, or a score less than 23 on the Mini-mental State Exam (Folstein et al., 1975). All participants provided oral and written consent prior to beginning the study. Prior to data collection, this study was approved by the Kennesaw State University Institutional Review Board. Self-reported physical activity was obtained with the International Physical Activity Questionnaire (Hagströmer et al., 2006).

### 2.2 Experimental design

**FIGURE 1:**
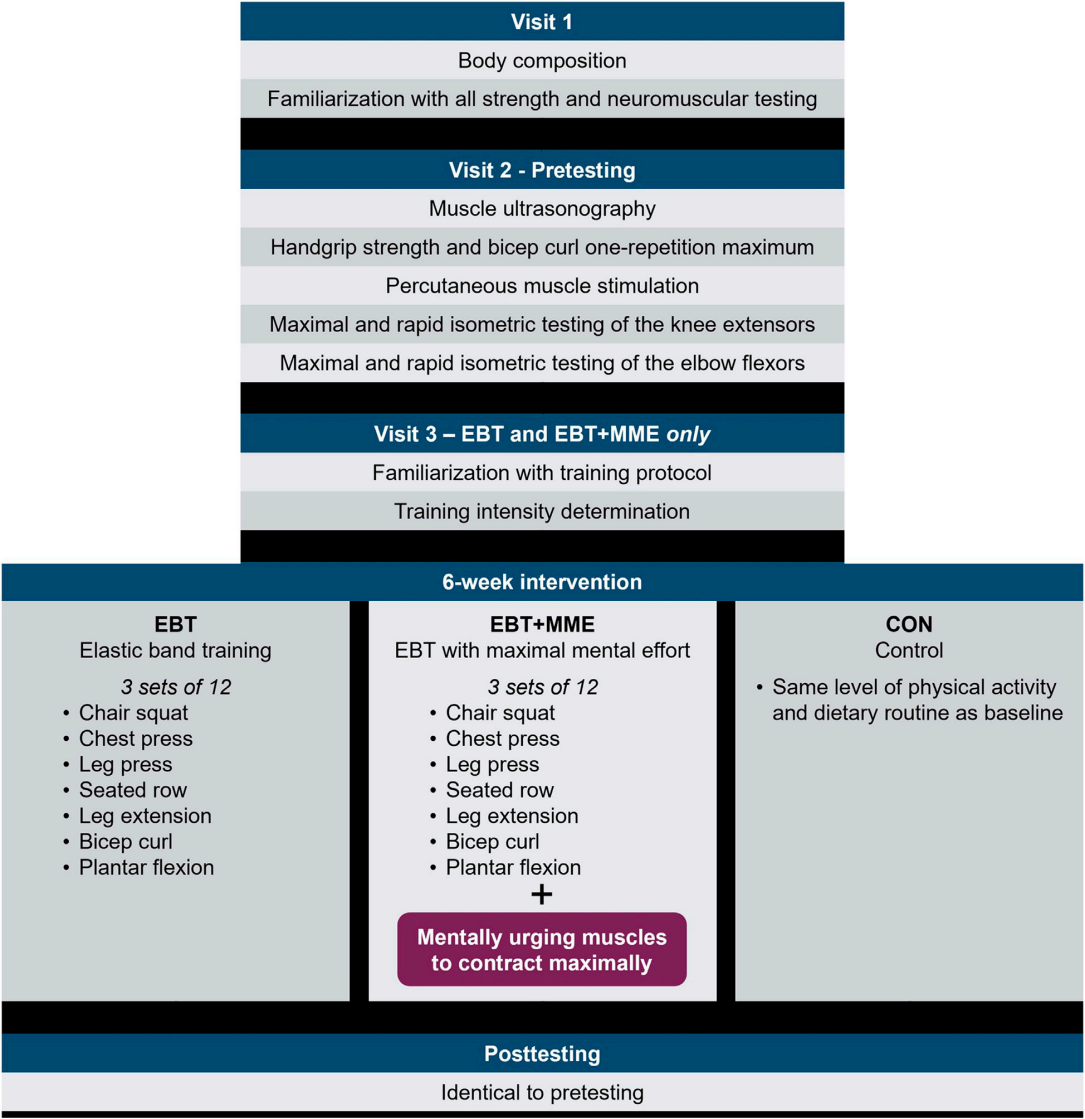
Study overview.

This randomized controlled trial involved three groups: elastic band training (EBT); elastic band training with MME (EBT + MME); and a control (CON) group. An overview of the study design is provided in Figure 1. The trial was preregistered at clinicaltrials.gov (NCT06627491). Participants were assigned to groups using block randomization (blocks of three) via an online random number generator. EBT performed 6 weeks of moderate-intensity RT with elastic bands, while EBT + MME performed the same training but with intentional MME during each contraction. CON participated in testing visits only, and all groups were instructed to maintain their normal physical activity and dietary habits. The study’s overarching purpose was concealed to participants, and specifically they were advised “the purpose was to determine the efficacy of a home-based RT program with elastic bands for older adults”. Prior to the intervention, all participants completed testing familiarization and pretesting across two lab visits. Additionally, participants in EBT and EBT + MME attended a third visit for training familiarization, which included MME instruction for EBT + MME. Posttesting (same as pretesting) was completed 3–7 days following the 6-week intervention. Participants were instructed to complete a 4-h fast prior to the first and final visit and to avoid alcohol 24 h prior to all visits. Home-based training sessions were held for individual participants and virtually supervised via a video chat platform by a trained member of the research team.

### 2.3 Training protocol

Each training session started with 5 min of dynamic stretches followed by EBT. All exercises were performed with TheraBand resistance bands (TheraBand®, Akron, OH, USA). Exercises were performed at moderate intensity indicated by a rating of perceived effort of 5–6 (somewhat hard) on the OMNI-Resistance Exercise Scale with elastic bands (Supplementary Figure S2), which has been validated for assessing perceived exertion in older adults (Colado et al., 2018). Training intensity determination followed a similar protocol to others using EBT (Colado et al., 2012) (Supplementary Section 2). At the beginning of the third and fifth weeks of the training period, the intensity determination protocol was repeated for each exercise to ensure moderate intensity was maintained.

The training regimen was performed in the same order by each participant and included both multi-joint and single-joint exercises (Figure 1). Participants in both training groups completed three training sessions per week for 6 weeks, with the exception of the first week in which only two training sessions were completed to minimize delayed onset muscle soreness. Participants performed three sets of 12 repetitions for each exercise with a 60-s rest interval between sets, and a similar cadence (1–2 s concentric and eccentric) was used by both groups.

### 2.4 Maximal mental effort

Those in EBT + MME underwent the same training protocol as EBT but were instructed to perform MME by mentally urging their muscles to contract maximally during each repetition. That is, despite using a moderate intensity with the elastic band, participants were instructed to *imagine* maximal effort by imagining the feeling (i.e., kinesthetic imagery) of maximal muscle contraction during the concentric portion of each repetition. Participants were instructed to focus the MME on the quadriceps femoris during the chair squat and leg press, latissimus dorsi during seated row, and the pectoralis major during chest press. For single-joint exercises, participants were instructed to focus the MME on the agonist muscle group. Prior to each set, participants in EBT + MME were consistently reminded to “imagine maximally contracting your muscle”, while participants in EBT were not given any additional instructions.

### 2.5 Affective feeling scale and qualitative questions

The Affective Feeling scale (Hardy and Rejeski, 1989) (Supplementary Figure S3) was used to determine how good/bad participants felt during the training sessions, and qualitative data was obtained during the posttesting session to assess participants’ focus of attention. Details of these assessments are presented in Supplementary Section 3.

### 2.6 Body composition

Height and body mass were measured using an electronic scale and stadiometer (Tanita WB 3000, Arlington Heights, IL, United States). Total body fat percentage and total, arm, and leg lean mass were obtained via bioelectrical impedance analysis (InBody770, InBody Co., Cerritos, CA, USA) following manufacturer recommendations.

### 2.7 Ultrasonography

Cross sectional area (CSA) and echo intensity (EI; indicator of muscle quality) (Pillen et al., 2009) of the vastus lateralis, rectus femoris, and biceps brachii (BB) were evaluated using a B-mode ultrasound (LOGIQ S7, General Electric Company, Milwaukee, WI, United States). Details on ultrasonography procedure are provided in Supplementary Section 4. EI was assessed via grey-scale analysis using the histogram function, where the mean was expressed as a value between 0 (black) and 255 (white). Subcutaneous fat thickness was measured and used to normalize EI as previously described (Young et al., 2015). CSA and EI of the vastus lateralis and rectus femoris were summed and averaged, respectively, to obtain quadriceps CSA and EI, and these values were used for statistical analysis.

### 2.8 One-repetition maximum and handgrip strength

A one-repetition maximum (1-RM) test for the bicep curl was performed while seated with the dominant limb using previously reported procedures (Rydwik et al., 2007). Handgrip strength was measured using a handgrip dynamometer (Jamar Plus Hand Dynamometer, Patterson Medical, Cedarburg, Wisconsin) while participants were seated with the elbow joint at approximately 90° and wrist in a neutral position. Three trials were performed, and participants were instructed to “squeeze as hard as possible” for approximately 5 seconds.

### 2.9 Electromyography and percutaneous muscle stimulation

Following skin preparation, surface electromyography (EMG) of the bicep brachii (BB) and rectus femoris were recorded at 2 kHz using parallel bar, bipolar surface electrodes (Delsys Trigno, Delsys, Inc., Natick, MA, USA) according to SENIAM recommendations. Neuromuscular assessments included percutaneous muscle stimulation of the BB followed by performance testing of the elbow flexors (EF) and knee extensors (KE) (muscle order randomized). The BB was electrically stimulated via a constant-current stimulator (Digitimer DS7AH, Welwyn Garden City, UK) with two surface electrodes arranged similar to previous work (Yoon et al., 2008). Initially, single 200 μs pulses were delivered at a low current (50 mA) and continually increased by 10–30 mA increments until the resting twitch plateaued. The stimulator current causing a plateau in resting twitch amplitude plus an additional 20% (pretesting: 232.2 ± 48.95 mA; posttesting: 231.52 ± 42.65 mA; p = 0.45) was used for the remainder of the protocol.

### 2.10 Maximal isometric strength and voluntary activation

Torque was sampled at 2 kHz during maximal voluntary isometric contractions (MVICs) of the dominant EF and KE using a calibrated Biodex four isokinetic dynamometer (Biodex Medical Systems, Inc. Shirley, NY, USA). The lateral epicondyle of the elbow and knee were aligned with the input axis of the dynamometer, and elbow and knee joint position were held at 90° during testing. Following a warm-up, participants performed two, 3–4 s MVICs separated by 2 minutes of rest. Additional trials were performed if peak torque (PT) from the first two trials varied by more than 5%. For the EF only, participants then performed another MVIC, during which a doublet stimulation (100 Hz) was delivered once PT plateaued (i.e., superimposed twitch) followed by a resting doublet stimulation delivered 2–3 s after the end of the contraction (i.e., potentiated twitch). Strong verbal encouragement and visual biofeedback were provided during all contractions.

### 2.11 Brief, rapid isometric contractions

Rate of torque development (RTD) was calculated from rapid (∼1 s), isometric contractions of the EF and KE using the same dynamometer under the instruction to “pull as fast and hard as possible” and “kick out as fast and hard as possible” for the EFs and KE, respectively. Five trials separated by 30 s were performed.

### 2.12 Neuromuscular data processing

The scaled, gravity corrected torque signals were digitally filtered with a zero lag, low-pass (50 Hz) Butterworth filter using custom written software (LabVIEW, National Instruments, Austin, TX). PT was considered the highest 500 m rolling average during MVIC testing prior to stimulation. RTD was calculated from the linear slope of the torque-time curve (Δtorque/Δtime) starting from a contraction onset of 1 Nm for the two rapid contractions with the highest peak RTD. RTD at its peak (rolling 10 m average), as well as from 0-50, 0-100, and 0–200 m were averaged across the two contractions. Absolute torque at 50, 100, and 200 m was also recorded.

Electromyography was processed using a fourth order Butterworth filter with a low- and high-frequency cutoff of 10 and 500 Hz, respectively, and applied to the scaled zero means signal. EMG amplitude was calculated as the root mean square for the 500 m epoch corresponding to peak torque (250 m either side) during MVICs. Rate of EMG rise (RER), a proxy for initial neural drive during explosive contractions (Klass et al., 2008), was determined from the smoothed EMG-time curve via a zero-lag 50 m moving root mean square. The onset was set at two SD above baseline EMG and the linear slope from onset to 50 m was calculated and then normalized to EMG amplitude from the MVIC for that testing visit. Contractile properties from the doublet delivered at rest included twitch PT and twitch peak RTD. Voluntary activation, an indicator of volitional neural drive during maximal effort, was assessed using the twitch interpolation technique (Merton, 1954) with a previously suggested factor suggested correction factor (Strojnik and Komi, 1998).

### 2.13 Statistical analyses

A total of 34 participants (EBT [*n* = 11], EBT + MME [*n* = 13], CON [*n* = 10]) completed the 6-week intervention (Figure 1), but the number of participants analyzed varied for quadriceps CSA and EI (*n* = 33), handgrip strength (*n* = 33), all EF torque outcomes (*n* = 33), contractile properties and voluntary activation (*n* = 30), and KE RER (*n* = 33) (Supplementary Section 1). Skewness and kurtosis values were divided by their standard error and a threshold of 1.96 was used to determine non-normality (Field, 2013). One-way ANOVAs were used to compare groups at baseline. Two-way mixed ANOVAs [group (EBT vs. EBT + MME vs. CON) × time (PRE vs. POST)] with group as a between-subjects factor were used to assess changes in body composition, ultrasound, strength, and neuromuscular outcomes. In the case of significant two-way interactions, follow-up ANOVAs comparing only EBT and EBT + MME across time were performed and pairwise comparisons were examined within each group. Bonferroni adjustments were applied to all follow-up tests. A three-way mixed ANOVA [group (EBT vs. EBT + MME vs. CON) × time (PRE vs. POST) × muscle (EF vs. KE)] with group as a between-subjects factor were used to assess changes in affective feelings. Levene’s test was used to assess homogeneity of variance. Handgrip strength, voluntary activation, and twitch peak RTD were non-normally distributed, and homogeneity of variance was unequal for quadriceps CSA. Given the smaller sample size for these outcomes and failure to meet an assumption of ANOVA, a non-parametric analysis was considered including the Kruskal–Wallis Test followed by Dunn’s Test. However, the findings were similar to parametric statistics. With the exception of quadriceps CSA, ANOVA findings are reported to maintain consistency and clarity. Partial eta squared $\left(\eta_{p}^{2}\right)$ was used for ANOVA analyses and <0.06, 0.07–0.14, and >0.14 indicated small, medium, and large effect sizes, respectively. Cohen’s *d* was used for pairwise comparisons and *r* was used for nonparametric tests with 0.20, 0.50, and 0.80, and 0.10, 0.30, and 0.50 indicating the same effect sizes, respectively (Cohen, 1988). All statistical analyses were performed with SPSS version 29 (IBM Corporation, Chicago, IL). An alpha level of p ≤ 0.05 was used to indicate statistical significance.

## 3 Results

### 3.1 Adherence, feelings about exercise, and focus of attention during interventions

The adherence to training for participants in the EBT and EBT + MME was 96.23% and 95.48%, respectively, and all participants completed at least 90% of their training sessions. No significant interaction (p > 0.05) was found for affective feeling indicating no differences between groups (Supplementary Table S1). Responses to the open-ended question, though qualitative, support the notion that a greater number of EBT + MME participants focused on the exercising muscle or effort (Supplementary Figure S4).

### 3.2 Group characteristics, body composition, muscle size, and muscle quality

Group characteristics and data for body composition, muscle size, and muscle quality with ANOVA findings are provided in Table 1. Total body and segmental lean mass remained unchanged (p > 0.05). Two-way interactions were found for BB CSA (p < 0.001; $\eta_{p}^{2}$ = 0.41), BB EI (p = 0.021; $\eta_{p}^{2}$ = 0.22), and quadriceps EI (p < 0.001; $\eta_{p}^{2}$ = 0.42). All outcomes were unchanged in CON (p > 0.05). BB CSA increased in in EBT (p < 0.001; *d* = 1.11) and EBT + MME (p < 0.001; *d* = 1.73) with no difference between groups (p = 0.392; $\eta_{p}^{2}$ = 0.03). BB EI decreased (improved quality) in EBT (p = 0.013; *d* = 0.86) and EBT + MME (p < 0.001; *d* = 0.89) with no difference between groups (p = 0.414; $\eta_{p}^{2}$ = 0.03). Quadriceps EI decreased in EBT (p < 0.001; *d* = 1.33) and EBT + MME (p < 0.001; *d* = 1.15) with no difference between groups (p = 0.669; $\eta_{p}^{2}$ = 0.009). Based on a significant Kruskal–Wallis Test (p < 0.001) and *post hoc* Dunn’s Test, quadriceps CSA increased similarly for EBT (p < 0.001; *r* = 0.88) and EBT + MME (p < 0.001; *r* = 0.88) compared to CON.

**TABLE 1:** Characteristics, body composition, and muscle size and quality for elastic band training (EBT), elastic band training with maximal mental effort (EBT + MME), and control (CON) groups.

	EBT		EBT + MME		CON		*p*-value	$\eta_{p}^{2}$
Variable	Pre	Post	Pre	Post	Pre	Post		
Age (yr)	70.55 ± 4.53	—	70.31 ± 3.97	—	71.30 ± 4.27	—	—	—
BMI (kg/m^2^)	28.95 ± 5.77	—	26.39 ± 4.49	—	29.03 ± 5.90	—	0.400^a^	0.06
MET (hrs/wk)	116.62 ± 58.42	—	102.97 ± 41.07	—	111.04 ± 70.46	—	0.838^a^	0.01
Body mass (kg)	77.44 ± 17.79	73.02 ± 22.97	68.80 ± 13.62	67.99 ± 11.94	72.94 ± 12.41	72.37 ± 11.85	0.463^b^	0.05
Body fat %	42.16 ± 9.05	41.71 ± 9.00	36.92 ± 7.95	36.76 ± 7.40	41.67 ± 8.05	41.47 ± 7.58	0.853^b^	0.01
Total lean Mass (kg)	11.56 ± 1.89	11.64 ± 1.80	11.38 ± 1.47	11.34 ± 1.46	11.10 ± 1.10	11.07 ± 1.03	0.481^b^	0.01
Arm lean Mass (kg)	1.03 ± 0.26	1.05 ± 0.25	0.89 ± 0.14	0.89 ± 0.15	0.95 ± 0.15	0.96 ± 0.14	0.696^b^	0.02
Leg lean Mass (kg)	2.85 ± 0.59	2.84 ± 0.56	2.84 ± 0.64	2.82 ± 0.58	2.72 ± 0.29	2.68 ± 0.26	0.753^b^	0.02
BB CSA (cm^2^)	5.28 ± 1.48	5.61 ± 1.51**	4.35 ± 0.99	4.78 ± 1.01**	4.57 ± 0.88	4.57 ± 0.86	<0.001^c^	0.41
BB EI (a.u.)	123.34 ± 22.71	118.28 ± 20.28*	111.97 ± 14.15	104.39 ± 12.87*	116.74 ± 11.47	117.05 ± 10.45	0.021^c^	0.22
Quadriceps CSA (cm^2^)	20.66 ± 5.67	22.05 ± 5.89^¥^	18.46 ± 2.86	19.47 ± 2.72^¥^	19.68 ± 3.35	19.59 ± 3.28	—	—
Quadriceps EI (a.u.)	155.41 ± 22.37	149.25 ± 22.10*	147.78 ± 14.39	142.47 ± 11.35*	150.04 ± 15.39	151.45 ± 15.01	<0.001^c^	0.42

MET, metabolic equivalent of task; BB, biceps brachii; CSA, cross-sectional area; EI, echo intensity.

^a^Similar between groups at baseline.

^b^Non-significant two-way interaction. Non-significant main effect for time (p > 0.05).

^C^Significant two-way interaction.

*Significant change from pre (p < 0.05).

^¥^Significant increase from pre via Kruskal–Wallis and Dunn’s test (p < 0.05).

### 3.3 Maximal isometric and dynamic strength

**FIGURE 2:**
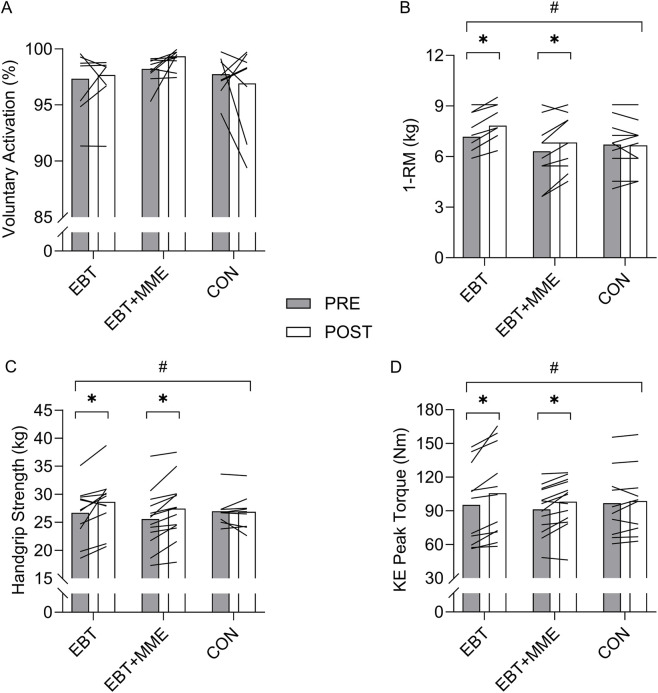
Voluntary activation **(A)**, bicep curl 1-RM **(B)**, handgrip strength **(C)**, and knee extensor (KE) peak torque **(D)** before and after elastic band training (EBT), elastic band training with maximal mental effort (EBT + MME), and control (CON). #indicates two-way interaction (p < 0.05).*indicates increase from pre (p < 0.05).

Data for all maximal isometric and dynamic strength measures with ANOVA findings and are provided in Table 2. Two-way interactions were found for bicep curl 1-RM (p = 0.004; $\eta_{p}^{2}$ = 0.29), KE PT (p = 0.034; $\eta_{p}^{2}$ = 0.20), and handgrip strength (p = 0.021; $\eta_{p}^{2}$ = 0.23). All outcomes were unchanged in CON (p > 0.05) (Figure 2). Bicep curl 1-RM increased in EBT (p < 0.001; *d* = 2.11) and EBT + MME (p < 0.001; *d* = 0.90) with no difference between groups (p = 0.493; $\eta_{p}^{2}$ = 0.02). KE PT increased in in EBT (p < 0.001; *d* = 1.14) and EBT + MME (p = 0.001; *d* = 1.16) with no difference between groups (p = 0.278; $\eta_{p}^{2}$ = 0.05). Handgrip strength increased in in EBT (p < 0.001; *d* = 0.83) and EBT + MME (p < 0.001; *d* = 1.39) with no difference between groups (p = 0.905; $\eta_{p}^{2}$ = 0.001).

**TABLE 2:** Maximal isometric and dynamic strength measures for elastic band training (EBT), elastic band training with maximal mental effort (EBT + MME), and control (CON).

	EBT		EBT + MME		CON		*p*-value^a^	${\;\eta }_{p}^{2}$
Variable	Pre	Post	Pre	Post	Pre	Post		
Bicep curl 1-RM (kg)	7.18 ± 1.16	7.83 ± 1.09**	6.31 ± 1.64	6.84 ± 1.50**	6.71 ± 1.60	6.67 ± 1.47	0.004^#^	0.299
Elbow flexors PT (Nm)	29.25 ± 7.53	29.64 ± 6.26	27.63 ± 5.86	28.22 ± 4.97	27.17 ± 3.71	25.68 ± 4.83	0.153	0.117
Knee extensors PT (Nm)	95.30 ± 35.10	105.53 ± 40.15**	91.25 ± 21.32	98.08 ± 22.14**	96.69 ± 30.57	98.62 ± 30.17	0.034^#^	0.195
Handgrip Strength (kg)	26.69 ± 4.74	28.64 ± 4.90**	25.58 ± 5.10	27.44 ± 5.26**	26.98 ± 2.74	26.87 ± 3.16	0.021^#^	0.227

1-RM, one-repetition maximum; PT, peak jtorque.

^a^p-value for two-way interaction.

#Significant two-way interaction.

**increased from pre (p ≤ 0.001; *d* = 0.90–2.11).

### 3.4 VA and twitch properties

Data for VA and all twitch properties with ANOVA findings are provided in Supplementary Table S2. No two-way interaction was found for VA (p = 0.182; $\eta_{p}^{2}$ = 0.119) (Figure 2), but the moderate effect size and a qualitative assessment of Figure 2 are noteworthy. For example, 83% and 67% of participants in EBT + MME and EBT, respectively, increased VA. All twitch properties remained unchanged (p > 0.05).

### 3.5 Rapid torque production and muscle activation

Data for all rapid torque measures and RER with ANOVA findings are provided in Supplementary Table S3. KE RTD 0–200 m exhibited a main effect for time (p = 0.026; $\eta_{p}^{2}$ = 0.166) which was largely driven by similar increases in EBT and EBT + MME. All other rapid torque measures and RER remained unchanged for all groups (p > 0.05).

## 4 Discussion

The purpose of this study was to determine if combining MME with EBT augments neuromuscular adaptations in older women. To the best of our knowledge, this is the first study to examine the potential synergistic effects of MME on neuromuscular outcomes when combined with practical, total-body resistance training. The current study expanded on previous work that was limited to electroencephalographic recordings by examining the effect of MME on VA, or the ability of the central nervous system to fully activate skeletal muscle. A short-term resistance exercise model was used to emphasize neural adaptations (i.e., VA) contributing to increases in strength. Neither strength nor VA was significantly augmented by MME, despite consistent individual level increases in VA for the EBT + MME group. It is unclear if the effect of MME was too small, or the present study was too underpowered to attain statistical significance. Regardless of MME, further evidence was demonstrated for the effectiveness of EBT for improving muscle size, quality, and strength.

The current study failed to find a synergistic effect of MME on VA when combined with moderate-intensity EBT in older women. Although statistical significance was absent, qualitative assessment of the individual changes in VA (Figure 2) indicates that far more EBT + MME participants demonstrated improvements than EBT or CON. It is also noteworthy that such consistent individual level increases were seen with baseline values already being quite high (discussed below). It is unclear whether MME poses an insignificant effect on VA or if the underpowered analysis was unable to detect the effect. Previously it was shown that MME combined with isometric exercise increased motor activity-related cortical potentials (Jiang et al., 2016), which indicates neuroplastic changes in higher-order cortical centers, but does not give indication of whether descending efferent drive is affected. Our findings suggest that MME may not increase the ability of the nervous system to activate skeletal muscle, but there are two major caveats to this notion. Firstly, this was an underpowered study, and these preliminary findings should stimulate enthusiasm for larger randomized controlled trials. Secondly, the current sample of older women already exhibited a high (complete) VA level (≥95%), as previously seen in other older adult samples (Allman and Rice, 2001; Klein et al., 2001). While not necessarily surprising since all participants were non-sarcopenic, physically active (based on self-reports), and under 80 years of age–There was little capacity for VA to improve since its values theoretically can only reach 100%. Given the moderate effect size, albeit non-significant, perhaps MME has a more dramatic effect on VA when baseline levels are lower in populations with greater functional decline. We posit this notion as a critical next step for future research. We also examined the effect of MME on RER, but no changes were demonstrated. It is reasonable to assume that, because the EBT involved a moderate load and no explosive intent, the MME supplementation did not induce any specific adaptations for explosive neuromuscular performance (Del Vecchio et al., 2024).

It was hypothesized that strength improvements would be augmented in the EBT + MME group due to greater neural adaptations, but improvements in handgrip strength, bicep curl 1-RM, and KE isometric strength were similar for EBT and EBT + MME. As previously described, given the high VA levels at baseline for our sample any further increase would likely not be meaningful towards strength (Rozand et al., 2020). Previously, in older adults, similar strength increases were found for a low-intensity isometric RT group that simultaneously used MME compared to a high-intensity group that watched a documentary while training (Jiang et al., 2016). While those findings suggest MME may augment strength increases that are typically minimal following low-intensity exercise (Peterson et al., 2010), uncertainty remains regarding the effectiveness of MME when employed with total body, dynamic exercise. Our findings are in line with previous literature which supports that moderate-intensity EBT provides a sufficient stimulus to improve strength in older adults (Colado and Triplett, 2008; Liao et al., 2018). These results support EBT as a practical RT modality for older adults, which is particularly desirable for those who experience greater functional decline or are not comfortable with conventional RT. Isometric peak torque of the EF was the only strength outcome that was unchanged after training. This finding may be explained by contraction type specificity on testing performance and more dramatic age-related loss of lower body muscle mass and function exhibited in women (Jones et al., 2021; Hunter et al., 2000; Janssen et al., 2000), where adaptations from dynamic, variable-load training were not reflected in upper body isometric testing. Finally, we also examined changes in rapid torque capacity, given it is predominantly affected by neural factors and its relevance for physical function in older adults (Hester et al., 2021; Del Vecchio et al., 2019). Aside from RTD 0–200 m (main effect for time), all other rapid torque outcomes were unchanged in the present study which is in line with mixed findings following slow- or moderate-velocity RT (Del Vecchio et al., 2022; Balshaw et al., 2016; Blazevich et al., 2008). The lack of improvement in early phase rapid torque outcomes is likely explained by the specific motor unit adaptations behind maximal force versus maximal speed production (Del Vecchio et al., 2024). Our strength findings add to numerous other studies regarding the efficacy of short-term EBT for increasing upper and lower body strength in older women (Herda and Nabavizadeh, 2021; Fritz et al., 2018). Given the commonly reported barriers to conventional RT for older adults, which include perceived difficulty, lack of time, and financial cost of health clubs (Burton et al., 2017; Van Roie et al., 2015), it is crucial to promote a low-cost, accessible methods of RT such as EBT to combat age-related strength loss.

Similar to our hypothesis, improvements in muscle size and quality were comparable between EBT and EBT + MME, with no changes in lean body mass. Surprisingly, studies examining muscle hypertrophy responses, exclusive of body composition, to EBT in older adults is scant. Our findings indicate the potential for muscle size increases in as little as 6 weeks with EBT, which is consistent with another study using conventional RT in older adults (Scanlon et al., 2014). Muscle quality, as indicated by ultrasonography, was also improved in both training groups which is similar to other short-term RT work including an EBT study (Fukumoto et al., 2014) and findings following conventional RT (Scanlon et al., 2014). While it is unclear why lean mass measures remained unchanged, previous research has demonstrated increased muscle CSA with no change in lean mass following short-term RT (Scanlon et al., 2014), and it is expected that longer term training would alter this result. Although the focus of the current work was on changes in neuromuscular and strength outcomes, the improvements in muscle size and quality, particularly after only 6 weeks, underscore the effectiveness of EBT for a variety of muscle health benefits.

There were several limitations associated with this study. The small sample size led to underpowered analyses and may not fully represent the sampled population. Nonetheless, the preliminary findings are important for future research considering MME as a cognitive strategy to mitigate age-related weakness. Additionally, while we presented qualitative data supporting efficacy in our MME group for their focus of attention, an inherent challenge with our cognitive intervention is the uncertainty that all participants adhered to the instructions. While participants were instructed to maintain their normal physical activity and dietary habits, tracking was not performed to ensure no changes occurred. Finally, a limitation of EBT is the inability to precisely quantify exercise volume and ensure equality between groups, but it should be noted that the protocol used to determine exercise intensity was validated for elastic bands in older adults.

This study did not find evidence of augmented strength or voluntary activation of skeletal muscle when a novel cognitive strategy, MME, was employed during 6-week of EBT. However, given the already high levels at baseline, the consistent individual level increases in voluntary activation are noteworthy enough to provoke further research on the efficacy of MME. An important consideration for future research is the functional status of the sample since an effect of MME, if any, should be stronger in sarcopenic or frail older adults with significant age-related weakness. The positive perceptions towards EBT with MME are noteworthy and indicate the additional cognitive demand of MME may not negatively affect exercise adherence. Finally, this study provided further evidence for the positive effects of home-based EBT on muscle strength, size, and quality in older women. Given the low rates of participation in resistance training by older adults (Keadle et al., 2016), promotion of accessible forms of resistance training such as EBT is critically important.

## Data Availability

The raw data supporting the conclusions of this article will be made available by the authors, without undue reservation.
