# Supplementary material for: Effect of maximal mental effort during elastic band training on neuromuscular adaptations in older women
Source: Front Aging. 2025 Nov 19;6:1662126. doi: 10.3389/fragi.2025.1662126 (PMC12672878; doi:10.3389/fragi.2025.1662126)
Supplement: Supplementary file 1 [file DataSheet1.pdf]

Supplementary Material

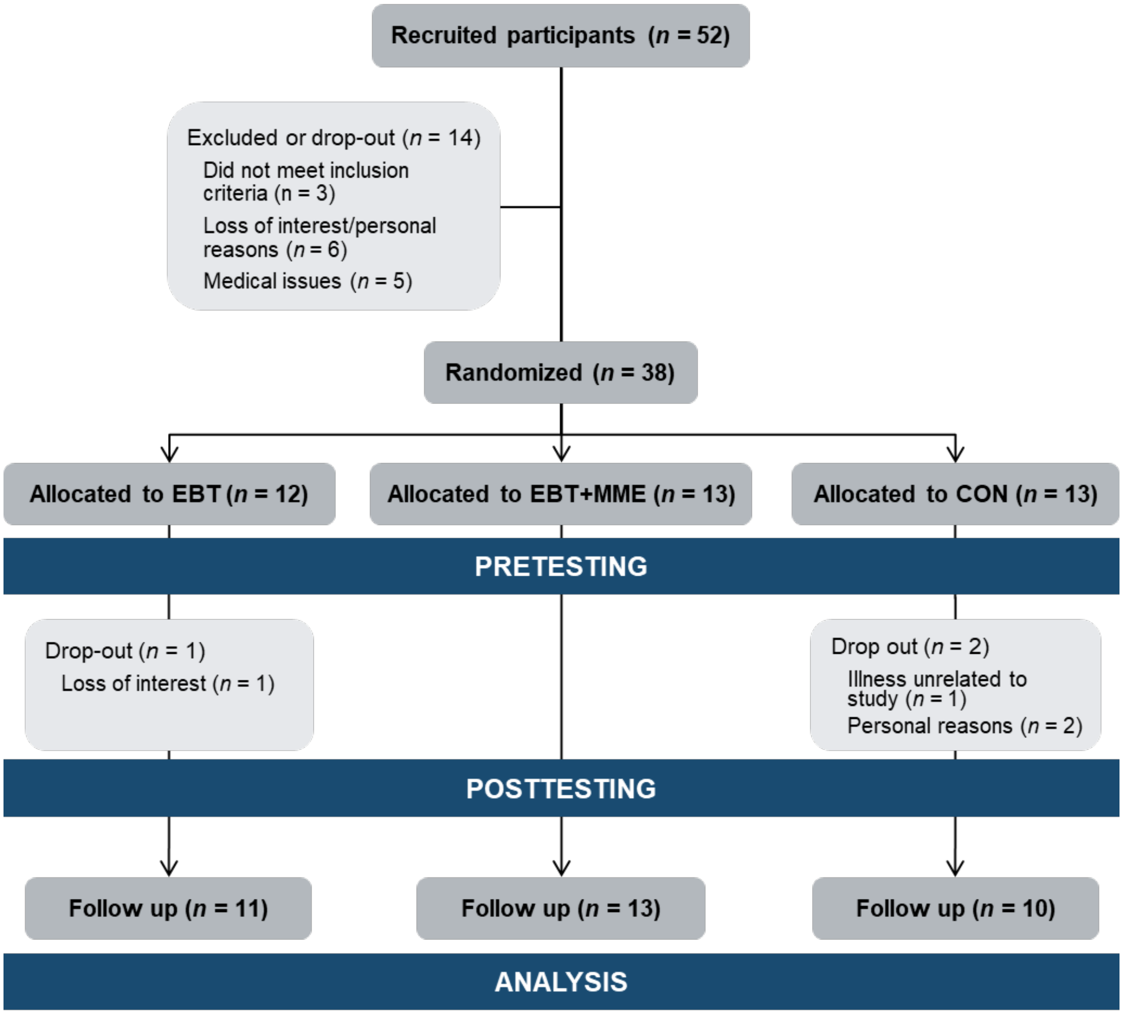

Figure S1. Study Participation flow diagram.

Section 1.

Total samples sizes used for statistical analysis were as follows: 33 participants for quadriceps CSA and EI due to presence of a metal implant (n = 1), 33 participants for handgrip strength due to pain unrelated to the study (n = 1), 33 participants for all EF torque outcomes due

to participant time constraints (*n* = 1), 30 participants for voluntary activation and contractile properties due to unwillingness to undergo stimulation (*n* = 4), and 33 participants for KE RER due to technical error (*n* = 1).

Section 2: Protocol for determining exercise intensity with elastic bands.

The Theraband® elastic bands were cut into 5-foot lengths then labelled and numbered symmetrically from the center for reproducibility of grip width. During the familiarization visit, participants were trained in the technique of each exercise then asked to perform several sets using an elastic band at minimum resistance to ensure proper execution. Then, participants performed each exercise at an estimated grip width on the elastic band that would allow them to maximally perform a total of 15 repetitions (15-RM). Multiple attempts were performed by widening the grip 1-2 increments each trial until the 15-RM was achieved. There was a 2-minute break between attempts. Participants were instructed to associate the effort following the 15-RM set as a "10" on the OMNI-RES (Figure S2). That is, their 15-RM for each exercise was considered maximal intensity. Moderate intensity (i.e., a 5-6 on the OMNI-RES scale) was achieved with a grip width that was 25% greater than the 15-RM width, which was utilized as the initial grip width for each exercise at the beginning of the training. Specifically, the initial training grip width was approximately 25% further from the center point of the elastic band compared to the grip width during the 15-RM.

).

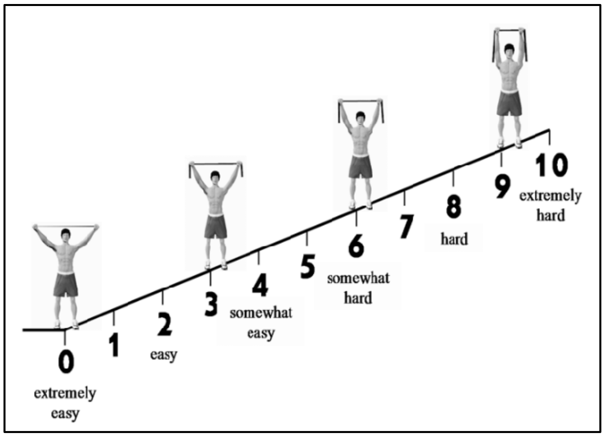

Figure S2. OMNI-Resistance Exercise Scale (OMNI-RES) for Theraband® elastic bands.

Section 3: Use of the Affective Feeling Scale and qualitative questions during and following training.

The Affective Feeling Scale (Figure S3) was used to determine how good/bad participants felt based on the following questions. Participants were asked “How did you generally feel after the knee extension/bicep curl exercise?” after those respective exercises and “How did you generally feel about that exercise session?” immediately after completion of each training session. Qualitative data, in the form of a single open-ended question, was obtained during the post-testing session. Specifically, “What did you typically think about while actively performing the exercises?” was asked of all training group participants. All responses were recorded and categorized based on recurring key terms (e.g., “muscle,” “form”), and the number of responses per category within each group were tallied. These frequencies were then visually represented as proportionally scaled bubbles, where the size of each bubble directly corresponded to the number of participants in a group who gave that type of response (Figure S4).

FEELING SCALE	
+5	Very Good
+4	
+3	Good
+2	
+1	Fairly Good
0	Neutral
-1	Fairly Bad
-2	
-3	Bad
-4	
-5	Very Bad

Figure S3. Affective Feeling Scale.

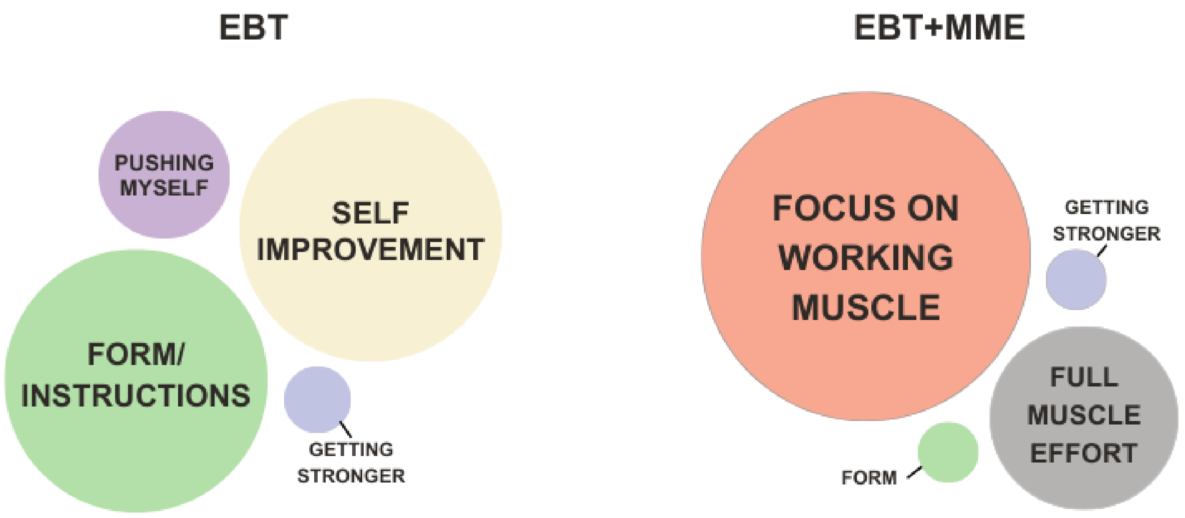

Figure S4. Keywords of responses to the question "What did you typically think about while actively performing the exercises?" for elastic band training (EBT) and with maximal mental effort (EBT+MME).

Table S1. Weekly Affective Scale results for elastic band training (EBT) and elastic band training with maximal mental effort (EBT+MME).

Group	Week 1	Week 2	Week 3	Week 4	Week5	Week 6	*p*-value	η_p^2
**EBT**	4.41 ± 0.66	4.58 ± 0.52	4.70 ± 0.41	4.80 ± 0.32	4.79 ± 0.34	4.91 ± 0.30	0.081[a]	0.103
**EBT+MME**	4.73 ± 0.33	4.62 ± 0.68	4.67 ± 0.59	4.64 ± 0.69	4.71 ± 0.60	4.64 ± 0.81		

[a]Non-signifcant two-way interaction

Section 4: Procedure for muscle ultrasonography.

Panoramic images were acquired with a 12-MHz linear-array probe (ML6-15 L; 5–13 MHz; 50 mm field of view; General Electric Company, Milwaukee, WI, United States) using the LogiqVIEW function. All images were taken by the same investigator with participants in the supine position. Participants rested in the supine position for 10 minutes prior to the images being captured. Three images were collected at following sites and settings: (1) 50% the distance from the greater trochanter to lateral femoral epicondyle with the knee fully extended (gain = 58 dB, depth = 6 cm) for the VL; (2) 50% the distance from the anterior superior iliac spine to the superior border of the patella with the knee fully extended (gain = 58 dB, depth = 6 cm) for the RF (1); (3) 66% of the distance from the medial acromion process to the cubital fossa with the shoulder abducted (gain = 58 dB, depth = 4 cm) for the BB (2). Ultrasound depth for the VL and RF was adjusted to 7 cm when necessary, due to subcutaneous fat thickness and kept consistent for posttesting. Thick, double-sided tape was placed over each muscle in the transverse plane to ensure the probe was moved perpendicular to the skin. Images were scaled from pixels to centimeters prior to analysis. CSA and EI of each muscle were determined using the polygon

function in ImageJ software (version 1.46r, National Institutes of Health, Bethesda, MD, United States) to select as much of the muscle as possible without including the surrounding fascia.

Section 5. Results for voluntary activation and twitch properties (Table S2) and rapid torque outcomes (Table S3).

Voluntary Activation and Twitch Properties

Table S2. Voluntary activation and twitch properties for elastic band training (EBT), elastic band training with maximal mental effort (EBT+MME), and control (CON).

	EBT		EBT+ME		CON			
**Variable**	**Pre**	**Post**	**Pre**	**Post**	**Pre**	**Post**	***p*-value**[a]	η_p^2
**Voluntary Activation (%)**	97.34 ± 2.87	97.66 ± 2.64	98.20 ± 1.17	99.29 ± 0.86	97.75 ± 1.77	96.90 ± 3.85	0.182	0.119
**Twitch PT (Nm)**	8.36 ± 2.02	8.56 ± 1.77	6.31 ± 1.93	6.65 ± 1.55	7.56 ± 1.73	6.96 ± 2.00	0.306	0.084
**Peak twitch RTD ($Nm \cdot s^{-1}$)**	344.79 ± 104.44	330.53 ± 83.91	280.47 ± 76.21	286.34 ± 103.24	327.51 ± 98.45	273.79 ± 86.58	0.236	0.102

PT = peak torque, RTD = rate of torque development, EF $RER_{0\text{-}50}$ = Elbow flexors rate of EMG rise at 0-50 ms.
[a]p-value for two-way interaction.

Rapid Torque Production and Muscle Activation

Table S3. Rapid torque outcomes for elastic band training (EBT), elastic band training with maximal mental effort (EBT+MME), and control (CON).

	EBT		EBT+ME		CON			

Variable	Pre	Post	Pre	Post	Pre	Post	*p*-value[a]	η_p^2
**EF Torque at 50 ms (Nm)**	13.05 ± 4.53	12.53 ± 3.67	9.71 ± 2.84	10.51 ± 3.13	11.04 ± 3.12	10.92 ± 2.80	0.357	0.066
**EF Torque at 100 ms (Nm)**	20.32 ± 7.29	20.42 ± 5.59	16.92 ± 4.70	17.51 ± 4.77	17.30 ± 3.49	16.78 ± 3.95	0.691	0.024
**EF Torque at 200 ms (Nm)**	24.18 ± 9.02	24.28 ± 6.67	22.00 ± 5.40	22.45 ± 5.38	21.74 ± 4.31	20.81 ± 4.90	0.642	0.029
**EF Peak RTD ($Nm{\cdot}s^{-1}$)**	376.99 ± 174.41	306.38 ± 102.17	243.48 ± 79.42	247.87 ± 66.69	285.57 ± 75.13	282.13 ± 83.70	0.134	0.125
**EF RTD 0-50 ms ($Nm{\cdot}s^{-1}$)**	266.83 ± 103.39	247.71 ± 82.36	182.56 ± 62.20	200.12 ± 66.29	216.78 ± 73.86	217.19 ± 68.02	0.222	0.095
**EF RTD 0-100 ms ($Nm{\cdot}s^{-1}$)**	213.04 ± 82.62	217.26 ± 63.01	176.19 ± 55.58	181.31 ± 52.82	179.05 ± 41.32	170.70 ± 44.90	0.639	0.029
**EF RTD 0-200 ms ($Nm{\cdot}s^{-1}$)**	112.31 ± 43.30	114.60 ± 33.81	106.98 ±9.58	107.13 ± 27.22	100.85 ± 25.75	95.79 ± 28.09	0.659	0.027
**EF RER ($\%RMS^{-1}$)**	19.50 ± 12.57	17.56 ± 14.39	10.13 ± 4.63	10.81 ± 4.64	17.75 ± 10.25	13.10 ± 5.03	0.135	0.125
**KE Torque at 100 ms (Nm)**	48.91 ± 18.28	52.95 ± 21.45	40.08 ± 17.10	42.12 ± 15.88	45.94 ± 12.49	44.13 ± 14.69	0.173	0.107
**KE Torque at 200 ms (Nm)**	76.62 ± 29.55	82.31 ± 33.51	68.23 ± 20.22	71.95 ± 20.52	75.85 ± 23.11	75.35 ± 23.41	0.105	0.136
**KE Peak RTD ($Nm{\cdot}s^{-1}$)**	784.83 ± 257.72	810.82 ± 320.21	640.45 ± 212.68	679.80 ± 217.99	700.48 ± 180.36	693.93 ± 191.86	0.678	0.025
**KE RTD 0-50 ms ($Nm{\cdot}s^{-1}$)**	389.07 ± 181.62	427.94 ± 203.61	333.95 ± 225.44	315.74 ± 162.17	333.90 ± 125.98	376.45 ± 154.79	0.324	0.070
**KE RTD 0-100 ms ($Nm{\cdot}s^{-1}$)**	533.02 ± 201.34	581.02 ± 239.09	428.55 ± 200.72	454.82 ± 185.36	496.09 ± 139.79	480.78 ± 175.29	0.299	0.075
**KE RTD 0-200 ms ($Nm{\cdot}s^{-1}$)**	425.53 ± 172.30	454.79 ± 190.45*	372.08 ± 112.75	400.78 ± 120.23*	421.43 ± 127.88	412.53 ± 132.94*	0.060	0.166
**KE RER ($\%RMS^{-1}$)**	9.53 ± 2.38	11.09 ± 7.31	9.24 ± 5.67	8.01 ± 3.96	6.99 ± 2.46	8.26 ± 3.12	0.135	0.125

KE = knee extensors; EF = elbow flexors; RTD = rate of torque development; RER = rate of electromyographic rise.

[a]p-value for two-way interaction.

*Significant main effect for time (p=0.026).

Supplementary Materials References

1. Hester GM, VanDusseldorp TA, Ha PL, Kiani K, Olmos AA, Jabbari M, et al. Microbiopsy Sampling for Examining Age-Related Differences in Skeletal Muscle Fiber Morphology and Composition. Frontiers in physiology. 2022;12:2385.

2. Stratton MT, Tinsley GM, Alesi MG, Hester GM, Olmos AA, Serafini PR, et al. Four weeks of time-restricted feeding combined with resistance training does not differentially influence measures of body composition, muscle performance, resting energy expenditure, and blood biomarkers. Nutrients. 2020;12(4):1126.
